# Nusinersen for children with type I spinal muscular atrophy: 4 years’ clinical experience in Turkish cohort

**DOI:** 10.3389/fneur.2025.1541507

**Published:** 2025-03-27

**Authors:** Ömer Bektaş, Murat Gülşen, Onur Burak Dursun, Ahmet Tekin, Deniz Yüksel, Ercan Demir, Gülten Öztürk, Sema Saltık, Özlem Hergüler, Ayşe Aysima Özçelik, Hüseyin Tan, Bilge Özgör, Arzu Ekici, Merve Feyza Yüksel, Süleyman Şahin, Özgür Duman, Mustafa Kömür, Figen Baydan, Edibe Pembegül Yıldız, Bülent Kara, Uluç Yiş, Seda Kanmaz, Kürşat Bora Çarman, Elif Acar Arslan, Mehmet Canpolat, Ahmet Sami Güven, Gökçen Öztuncer, Aycan Ünalp, Didem Ardıçlı, Aynur Ayşe Karaduman, Gökmen Zararsız, Gülhis Deda, Turkish SMA Study Group, Ömer Bektaş, Murat Gülşen, Onur Burak Dursun, Ahmet Tekin, Deniz Yüksel, Ercan Demir, Gülten Öztürk, Sema Saltık, Özlem Hergüler, Ayşe Aysima Özçelik, Hüseyin Tan, Bilge Özgör, Arzu Ekici, Merve Feyza Yüksel, Süleyman Şahin, Özgür Duman, Mustafa Kömür, Figen Baydan, Edibe Pembegül Yıldız, Bülent Kara, Uluç Yiş, Seda Kanmaz, Kürşat Bora Çarman, Elif Acar Arslan, Mehmet Canpolat, Ahmet Sami Güven, Gökçen Öztuncer, Aycan Ünalp, Didem Ardıçlı, Aynur Ayşe Karaduman, Gökmen Zararsız, Nurşah Yeniay Süt, Olcay Ünver, Tuğçe Damla Dilek, Eif Yıldırım, Faruk İncecik, Meral Karadağ, Mehmet Fatih Bütün, Burçin Gönüllü Polat, Yiğithan Güzin, Mehmet Akif Kılıç, Adnan Deniz, Gamze Sarıkaya Uzan, Sanem Yılmaz Keskin, Fatih M. Özdemir, Coşkun Yarar, Nihal Yıldız, Hamit Acer, Hüseyin Çaksen, Seren Aydın, Pakize Karaoğlu, Ayşe Neşe Çıtak, Tanıl Kendirli, Birkan Sonel Tur, Gülcan Akyüz, Ceren Bibinoğlu Amirov, Gülen Gül Mert, Mahmut Aslan, Şenay Haspolat, Özlem Ersoy, Osman Kipoğlu, Defne Alikılıç, Hasan Tekgül, Ülkühan Kaya, Arife Derda Yücel Şen, Pınar Özkan Kart, Hüseyin Per, Ayşe Aksoy, Ünsal Yılmaz, Serap Teber, Özben Akıncı Göktaş, Dilşad Türkdoğan, Fitnat Uluğ, Serap Bilge, Orhan Coşkun, Merve Öztürk, Esra Atmaca, Ali Cansu, Hakan Gümüş, Evrim Karadağ Saygı, Ömer Karaca, Serdal Güngör, Nazan Çobanoğlu, Melek Sezgin, Miraç Yıldırım, Yasemin Gökdemir, Esma Şengenç, Yılmaz Zindar, Özlem Yayıcı, Serkan Köken, Mesut Güngör, Serkan Kırık, Meltem Çobanoğulları Direk, Gülhis Deda

**Affiliations:** ^1^Division of Pediatric Neurology, Department of Pediatrics, Faculty of Medicine, Ankara University, Ankara, Türkiye; ^2^Ministry of Health, General Directorate of Health Services, Department of Autism, Mental Special Needs, and Rare Diseases, Ankara, Türkiye; ^3^Ministry of Health, General Directorate of Health Services, Ankara, Türkiye; ^4^Division of Pediatric Neurology, Department of Pediatrics, Dr. Sami Ulus Research and Training Hospital, University of Health Sciences Turkey, Ankara, Türkiye; ^5^Division of Pediatric Neurology, Department of Pediatrics, Faculty of Medicine, Gazi University, Ankara, Türkiye; ^6^Division of Pediatric Neurology, Department of Pediatrics, Faculty of Medicine, Marmara University, İstanbul, Türkiye; ^7^Division of Pediatric Neurology, Department of Pediatrics, Faculty of Medicine, İstanbul University Cerrahpaşa, İstanbul, Türkiye; ^8^Division of Pediatric Neurology, Department of Pediatrics, Faculty of Medicine, Çukurova University, Adana, Türkiye; ^9^Division of Pediatric Neurology, Department of Pediatrics, Faculty of Medicine, Gaziantep University, Gaziantep, Türkiye; ^10^Division of Pediatric Neurology, Department of Pediatrics, Faculty of Medicine, Atatürk University, Erzurum, Türkiye; ^11^Division of Pediatric Neurology, Department of Pediatrics, Faculty of Medicine, Turgut Ozal Research Center, Inonu University, Malatya, Türkiye; ^12^Division of Pediatric Neurology, Department of Pediatrics, Bursa Yüksek İhtisas Research and Training Hospital, University of Health Sciences Turkey, Ankara, Türkiye; ^13^Division of Pediatric Neurology, Department of Pediatrics, Faculty of Medicine, Akadeniz University, Antalya, Türkiye; ^14^Division of Pediatric Neurology, Department of Pediatrics, Faculty of Medicine, Mersin University, Mersin, Türkiye; ^15^Division of Pediatric Neurology, Department of Pediatrics, Tepecik Research and Training Hospital, İzmir, Türkiye; ^16^Division of Pediatric Neurology, Department of Pediatrics, İstanbul University, İstanbul, Türkiye; ^17^Division of Pediatric Neurology, Department of Pediatrics, Faculty of Medicine, Kocaeli University, Kocaeli, Türkiye; ^18^Division of Pediatric Neurology, Department of Pediatrics, Faculty of Medicine, Dokuz Eylül University, İzmir, Türkiye; ^19^Division of Pediatric Neurology, Department of Pediatrics, Faculty of Medicine, Ege University, İzmir, Türkiye; ^20^Division of Pediatric Neurology, Department of Pediatrics, Faculty of Medicine, Eskişehir Osmangazi University, Eskişehir, Türkiye; ^21^Division of Pediatric Neurology, Department of Pediatrics, Faculty of Medicine, Karadeniz Teknik University, Trabzon, Türkiye; ^22^Division of Pediatric Neurology, Department of Pediatrics, Faculty of Medicine, Erciyes University, Kayseri, Türkiye; ^23^Division of Pediatric Neurology, Department of Pediatrics, Faculty of Medicine, Necmettin Erbakan University, Konya, Türkiye; ^24^Division of Pediatric Neurology, Department of Pediatrics, Faculty of Medicine, Ondokuz Mayıs University, Samsun, Türkiye; ^25^Division of Pediatric Neurology, Department of Pediatrics, Faculty of Medicine, Dr. Behçet Uz Children’s Education and Research Hospital, University of Health Sciences Turkey Izmir, Izmir, Türkiye; ^26^Division of Pediatric Neurology, Department of Pediatrics, Ankara City Hospital, University of Health Sciences Turkey, Ankara, Türkiye; ^27^Department of Physiotherapy and Rehabilitation, Faculty of Medicine, Lokman Hekim University, Ankara, Türkiye; ^28^Department of Biostatistics, Erciyes University School of Medicine, Kayseri, Türkiye; ^29^Turkish Medicines and Medical Devices Agency, Ankara, Türkiye; ^30^Division of Intensive Care Unit, Department of Pediatrics, Faculty of Medicine, Ankara University, Ankara, Türkiye; ^31^Department of Physical Therapy and Rehabilitation, Faculty of Medicine, Ankara University, Ankara, Türkiye; ^32^Department of Physical Therapy and Rehabilitation, Faculty of Medicine, Marmara University, İstanbul, Türkiye; ^33^Division of Pediatric Pulmonology, Department of Pediatrics, Faculty of Medicine, Ankara University, Ankara, Türkiye; ^34^Department of Physical Therapy and Rehabilitation, Faculty of Medicine, Mersin University, Mersin, Türkiye; ^35^Division of Pediatric Pulmonology, Department of Pediatrics, Faculty of Medicine, Marmara University, İstanbul, Türkiye; ^36^Division of Pediatric Neurology, Department of Pediatrics, Faculty of Medicine, Selçuk University, Konya, Türkiye; ^37^Division of Pediatric Neurology, Department of Pediatrics, Elazığ Fethi Sekin City Hospital, Health Sciences University, Elazığ, Türkiye

**Keywords:** spinal muscular atrophy, SMA type 1, motor function, severe symptomatic, nusinersen, ventilatory, bulbar function, presymptomatic

## Abstract

**Background:**

SMA Type 1 is the most severe form of spinal muscular atrophy with early symptom onset, limited motor development, and poor prognosis. Recent genetic-based therapies, such as nusinersen, have transformed disease outcomes. We aimed to evaluate the long-term effects of nusinersen on motor, bulbar, and respiratory functions in both symptomatic and presymptomatic SMA Type 1 patients over a period of up to 4 years.

**Methods:**

This prospective, non-interventional study included 310 patients with genetically confirmed spinal muscular atrophy at 24 pediatric neurology centers in Turkey. Patients treated with nusinersen were divided into five age-based cohorts at treatment initiation: Cohort A (0–3 months), Cohort B (4–6 months), Cohort C (7–12 months), Cohort D (13–24 months), and Cohort E (>24 months). Efficacy was assessed using the CHOP-INTEND and WHO Motor Milestone Scale. This study also analyzed the respiratory support needs, gastrostomy requirements, and mortality rates across cohorts.

**Results:**

Patients treated before 12 months of age showed the most significant improvements in motor milestones, with 58.7% of Cohort A achieving independent sitting. CHOP-INTEND scores increased notably in all cohorts, with the largest improvement observed in Cohort A (93.5%). Ventilator and gastrostomy requirements decreased in the early treated cohorts. Adverse events were rare, with one discontinuation due to hydrocephalus. The overall mortality rate was 21.3%, with most of the deaths occurring within the first year.

**Interpretation:**

Nusinersen treatment initiated before 12 months of age, especially before 3 months of age, yielded the most favorable motor outcomes in patients with SMA type 1. Early initiation is associated with improved motor milestones and reduced need for ventilatory support. However, no significant improvements were observed in the bulbar function or in patients requiring extensive respiratory support.

## Introduction

The natural history of spinal muscular atrophy (SMA) has changed significantly owing to the introduction of novel therapeutics. The advent of spinraza (nusinersen) represents an effective treatment approach, and it became the first drug to be approved for SMA by the U.S. Food and Drug Administration (FDA) in December 2016 and the European Agency (EMA) in 2017 (1). With approximately 200 new cases diagnosed annually, Turkey has one of the highest SMA prevalence rates worldwide (2). Turkey was among the first countries to approve and reimburse for treatment.

Nusinersen, an antisense oligonucleotide, enhances the production of full-length SMN protein through modulation of SMN2 mRNA splicing (3). This leads to increased SMN protein levels, which can have significant disease-modifying effects in spinal muscular atrophy (SMA), potentially altering its natural course and improving motor function (4). Many studies, including data from early access programs, short-term real-world data, and clinical trials, have shown improvements in motor function and event-free survival in patients with SMA type 1 patients (5–15). However, long-term follow-up studies, particularly those involving both severely symptomatic and presymptomatic patients, remain limited (9).

During the orphan drug approval phase, limited information is available on the long-term effects of drugs in a wide range of individuals and data from a broad spectrum of patients are required to demonstrate the efficacy and safety of the drug. Here, we report data on the motor, bulbar, and respiratory effects of nusinersen in patients with symptomatic and presymptomatic early onset SMA for up to 4 years.

## Methods

### Study population

This non-interventional, single-arm, prospective follow-up study was conducted at tertiary pediatric neurology centers (n = 24) in Turkey and was approved by the coordinating center’s Ethics Committee and Regulatory Authority as per applicable local regulations.

Genetic analysis for the diagnosis of spinal muscular atrophy (SMA) was conducted using multiplex ligation-dependent probe amplification (MLPA) to assess the presence and copy number of the survival motor neuron 1 (SMN1) and survival motor neuron 2 (SMN2) genes. Genomic DNA was extracted from peripheral blood samples using a commercial DNA extraction kit following the manufacturer’s protocol. The presence of homozygous deletion of SMN1 exons 7 and 8, the primary genetic cause of SMA, was determined using SALSA® MLPA® Probemix P021 and P060 kits (MRC-Holland, Amsterdam, Netherlands), according to the standard protocol (16). After completing the consent process with the parents or legal guardians of all eligible subjects, genetically confirmed SMA patients with two or three copies of the SMN2 gene were enrolled in the study. None of the newborn screening programs were included in this study. No further data were collected after discontinuation of nusinersen if patients switched their medications. None of the patients concurrently received risdiplam or onasemnogene abeparvovec. Subjects who underwent tracheostomy and/or gastrostomy were defined as the subcohorts. The participants were stratified into five age-based cohorts according to treatment onset: Cohort A (0–3 months), Cohort B (4–6 months), Cohort C (7–12 months), Cohort D (13–24 months), and Cohort E (> 24 months).

### Data collection

The data for this study were collected from December 2017 until the end of April 2022, initially set data-cut-off date. Turkey’s regulatory authority guided the study design, site selection, and monitoring activities. All participating sites entered patient data through a web-based e-CRF into a validated database. The data were regularly checked by the data management team of the coordinating site and missing data were completed through queries.

### Evaluation methods of the study population

All the physiotherapists were trained by the same instructor (AAK). The primary efficacy criteria were set as the CHOP-INTEND and WHO motor milestone scales, and evaluations were performed by trained physiotherapists for motor assessment of patients. Independent sitting was defined as maintaining an upright posture with the head held high for a minimum of 10 s without any assistance from the hands or arms. The Childhood Health Assessment Questionnaire (CHOP INTEND) includes 16 components, resulting in a maximum score of 64, with higher scores indicating better motor skill. The physiotherapists participating in the study were regularly trained to ensure consistency in their assessments and achieve a high level of inter-rater reliability. We classified patients with SMA type I into type Ia (symptom onset before 1 month), type Ib (symptom onset between 1 month and 3 months), and type Ic (symptom onset between three and 6 months).

The study population was treated with Nusinersen according to the standard national protocol. The treatment regimen consisted of intrathecal administration of 12 mg (5 mL) Nusinersen, regardless of the patient’s age. The initiation phase included injections on days 0, 14, 30, and 60, followed by maintenance doses administered every 4 months.

The respiratory and nutritional status of the patients was regularly evaluated by a multidisciplinary team consisting of pediatric gastroenterologists, pediatric pulmonologists, and pediatric intensive care specialists. Respiratory assessments included clinical evaluation of cough strength, secretion management, and the need for invasive mechanical ventilation (IMV). Nutritional status was assessed through weight monitoring, body mass index (BMI) tracking, and caloric intake assessment. Furthermore, decisions regarding the initiation of gastrostomy or nasogastric tube feeding were made by a multidisciplinary team based on the patients’ nutritional status and swallowing function.

Hospital visits for nusinersen treatment and neurological examinations were recorded. A complete blood count, biochemistry panel, and urine analysis were performed before the application of each nusinersen dose. Respiratory parameters and nutritional status of each patient were monitored and recorded regularly.

CHOP-INTEND scores were evaluated at predetermined time points: baseline (T0), 6 (T6), 14 (T14), 26 (T26), 38 (T38), and 46 months (T46). The most recent CHOP-INTEND score recorded was designated as the post-treatment score to assess therapeutic efficacy.

### Statistical analysis

Histograms and q-q plots were constructed, and the Shapiro–Wilk test was conducted to assess the normality of the data. The Levene’s test was used to test variance homogeneity. To compare differences among the groups, Kruskal–Wallis H tests were used for continuous variables, and Pearson’s chi-square analysis or Fisher–Freeman–Halton test was used for categorical variables. Bonferroni-adjusted Dunn ‘sand Bonferroni adjusted *z-*tests were used for multiple comparisons. In addition, the Mc-Nemar and Mc-Nemar Bowker test statistics were used to compare feeding and respiratory changes before and after treatment. Univariate and multiple binary logistic regression analyses were applied to identify the risk factors for a 4-points increases. Significant variables at the *p* < 0.05 contingency level were included in multiple models, and backward elimination was performed using likelihood ratio statistics to identify the independent risk factors of rapid progression. The goodness-of-fit of the model was assessed using the Hosmer-Lemeshow test and Nagelkerke R2 statistic. Ordinary least-squares linear regression analyses were conducted to analyze the effect of the post-treatment CHOP-INTEND score. Kaplan–Meier curves were generated to estimate the survival probability of the age at the initiation of treatment, and the log-rank test was used for survival probability comparisons among the groups. In addition, the probabilities of gaining the ability to support sitting, gaining the ability to sit without support, need for ventilator in respiratory, and need for nasogastric tube or gastrostomy for feeding are presented as cumulative incidences. Analyses were conducted using R 4.2.0^1^ software. A *p*-value less than 5% was considered statistically significant.

## Results

### Demographic data

A total of 310 patients diagnosed with SMA type 1 at 24 clinical sites were enrolled in this study and received at least 4 doses of Nusinersen treatment. Among these, 50.3% (*n* = 156) were classified as type 1a, 38.7% (*n* = 120) as type 1b, and 11% (*n* = 34) as type 1c. The gender distribution was almost even, with a slight majority of males (52.3% vs. 47.7%). The mean age at symptom onset was 2.4 ± 1.1 months (range: 0–5) and a substantial percentage of patients had two copies of SMN2 (97.4%//n: 302). The mean age at the time of initiation of nusinersen treatment was 11.7 ± 18.3 months (range: 0–120, median: 5), while the median monitoring time was 14 (2–48) months. The mean CHOP-INTEND score of the patients was 16.7 ± 11.7 (median, 15; range: 0–57) at baseline (Table 1). Supplementary Table 1 provides the demographic characteristics of the cohorts according to age at treatment initiation. The number of patients per cohort and time points are listed in Supplementary Table 2.

**Table 1:** Demographic characteristics of SMA patients at baseline.

Distribution of patients-SMA type; *n* (%)	310 (100)
Type 1a	156 (50.3)
Type 1b	120 (38.7)
Type 1c	34 (11)
SMN copies; *n* (%)	
2 copies	302 (97.4)
3 copies	8 (2.6)
Gender	
Female; *n* (%)	162 (52.3)
Male; *n* (%)	148 (47.7)
Age at onset of SMA; mean + SD (range) in months	2.4 ± 1.1 (0–5)
Age at onset of treatment, median (range) in months	5 (1–120)
Monitoring time, median (range) in months	14 (2–48)
CHOP-INTEND score, at baseline; median (range)	16 (0–50)
CHOP-INTEND score, post-treatment; median (range)	28.0.5 (1–64)
Number of Nusinersen doses, median, (range)	5 (4–15)
Events of death during monitoring; *n* (%)	66 (21.3)
First year (%, within death)	(68.2)
Second year (%, within death)	(27.3)
Third year (%, within death)	(6.1)
Presymptomatic patients *n* (%)	7 (2.2)

### Motor milestones

**Figure 1:**
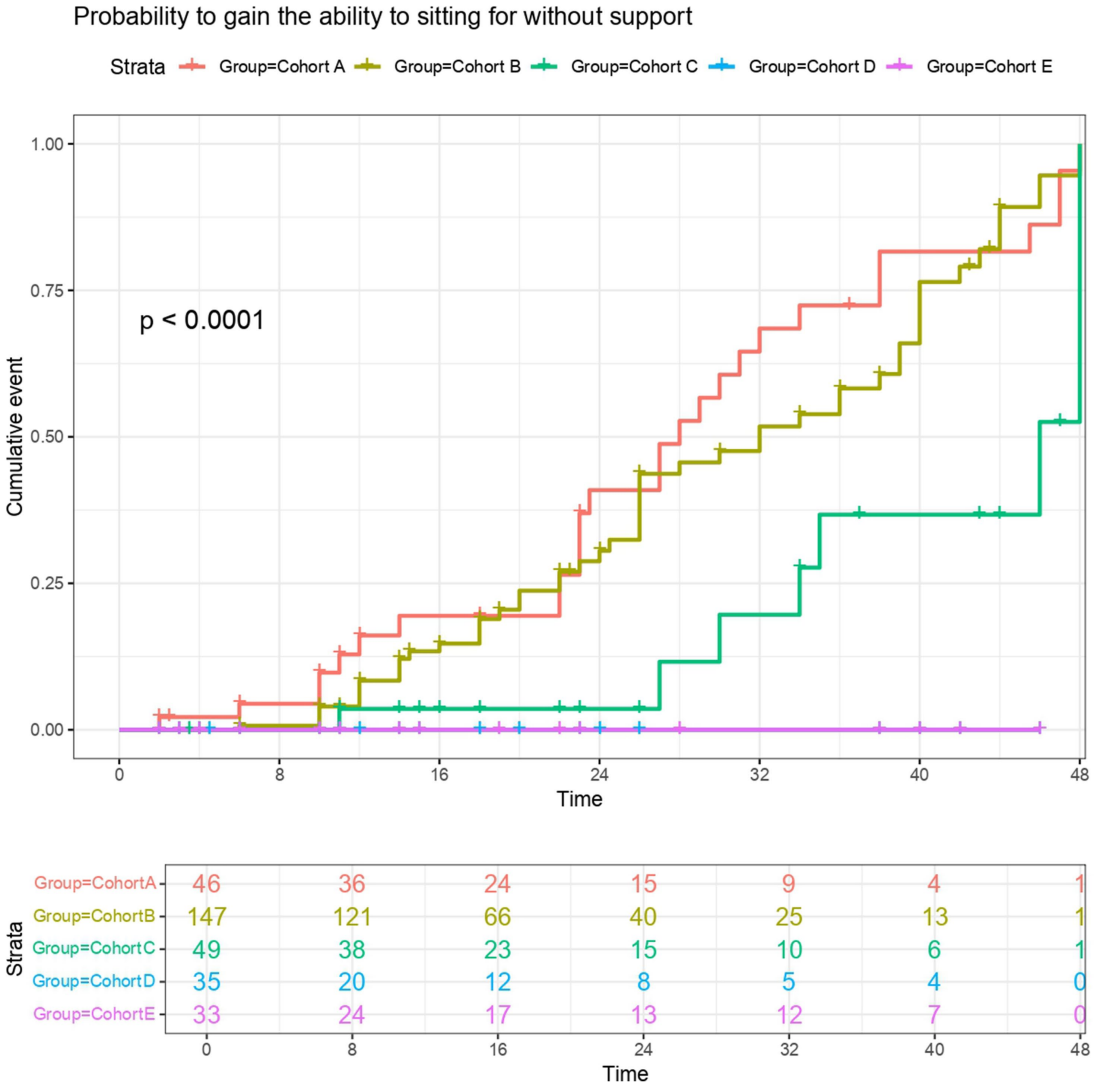
Probability of gaining the ability to sit without support. The probability of gaining the ability to sit without support in Cohort A (red), Cohort B (khaki), Cohort C (green), Cohort D (blue), and Cohort E (purple). Numbers at risk are listed for dedicated time points.

Following treatment, 58.7% (*n* = 27) of patients in cohort A, 34.7% (*n* = 51) in cohort B, and 14.3% (*n* = 7) in cohort C achieved the ability to sit unsupported. In cohort C, three of the seven patients who achieved unsupported sitting were classified as type 1b, while four were classified as type 1c. The most favorable motor milestone response was observed in Cohort A. In this cohort, three children (6.5%) acquired the ability to stand and eight children (17.4%) had the ability to walk. In contrast, cohort B had lower rates of standing (3.4%, *n* = 5) and walking (5.4%, *n* = 8). Table 2 summarizes the motor milestones achieved after nusinersen treatment, and Figure 1 illustrates the probability of acquiring the ability to sit unsupported across all the cohorts. None of the patients experienced loss of previously attained motor milestones following treatment. The cumulative incidence of achieving unsupported sitting was significantly higher in cohorts A and B than in the other cohorts and significantly higher in cohort C than in cohorts D and E (*p* < 0.0001). Supplementary Figure S1 shows the probability of achieving supported sitting across all the cohorts. Notably, supported sitting was not observed in any of the patients who began treatment after 13 months of age.

**Table 2:** Motor milestones.

Variable	The age at the initiation of treatment					Total (*n* = 310)	*p*- value
	Cohort A (*n* = 46)	Cohort B (*n* = 147)	Cohort C (*n* = 49)	Cohort D (*n* = 35)	Cohort E (*n* = 33)		
Post-treatment head control (%)	41 (89.1)^a^	112 (76.2)^a^	26 (53.1)^b^	11 (31.4)^bc^	4 (12.1)^c^	194 (62.6)	**<0.001**
Age at onset of treatment (months)	3.00 (2.50–3.01)	5.00 (4.00–5.50)	7.00 (6.99–7.50)	13.00 (12.00–14.50)	35.00 (28.75–45.00)		
Post-treatment supported sitting (%)	36 (78.3)^a^	91 (61.9)^a^	19 (38.8)^b^	2 (5.7)^c^	0 (0.0)^c^	148 (47.7)	**<0.001**
Age at onset of treatment (months)	3.00 (2.50–3.01)	5.00 (4.00–5.01)	7.00 (6.99–7.01)	13.00 (12.99–13.01)	–		
Post-treatment ability to rolling (%)	33 (71.7)^a^	71 (48.3)^a^	10 (20.4)^b^	0 (0.0)^c^	0 (0.0)^bc^	114 (36.8)	**<0.001**
Age at onset of treatment (months)	3.00 (2.50–3.01)	5.00 (4.00–5.50)	7.00 (6.99–7.13)	–	–		
Post-treatment sitting for short periods without support (%)	32 (69.6)^a^	65 (44.2)^b^	9 (18.4)^c^	0 (0.0)^c^	0 (0.0)^c^	106 (34.2)	**<0.001**
Age at onset of treatment (months)	3.00 (2.50–3.01)	5.00 (4.00–5.50)	7.00 (6.00–7.01)	–	–		
Post-treatment sitting for without support (%)	27 (58.7)^a^	51 (34.7)^b^	7 (14.3)^bc^	0 (0.0)^c^	0 (0.0)^c^	85 (27.4)	**<0.001**
Age at onset of treatment (months)	3.00 (2.50–3.01)	5.00 (4.00–5.50)	7.00 (6.99–7.01)	–	–		
Post-treatment standing with support (%)	13 (28.3)^a^	21 (14.3)^ab^	4 (8.2)^ab^	0 (0.0)^b^	0 (0.0)^b^	38 (12.3)	**<0.001**
Age at onset of treatment (months)	3.00 (2.25–3.01)	5.00 (4.00–5.01)	7.00 (6.99–7.01)	–	–		
Post-treatment standing without support (%)	8 (17.4)	8 (5.4)	1 (2.0)	0 (0.0)	0 (0.0)	17 (5.5)	0.001
Age at onset of treatment (months)	3.00 (2.13–3.01)	5.00 (4.63–5.75)	7.00 (6.99–7.01)	–	–		
Post-treatment walking (%)	3 (6.5)	5 (3.4)	0 (0.0)	0 (0.0)	0 (0.0)	8 (2.6)	0.175
Age at onset of treatment (months)	2.00 (1.99–2.00)	5.00 (4.50–5.50)	–	–	–		

SMA, Spinal Muscular Atrophy. Statistically significant values are in bold. Values are shown as *n* (%). In post hoc comparisons the same superscripts indicate similarities among groups, while different superscripts indicate a statistically significant differences among groups. Values are shown as *n* (%), mean ± standard deviation, or median (1st-3rd quartiles).

## Chop-intend

The median baseline CHOP-INTEND scores were 22 and 19 in cohorts A, B, 14 in cohort C, 9 in cohort D, and 6 in cohort E, respectively. After treatment, the scores increased to 43.5, 39, 20, 15, and 11 in cohorts A, B, C, D, and E, respectively (*p* < 0.001). The most notable and significant increase was observed in Cohort A, with the largest improvement observed at T14. The CHOP-INTEND scores across all cohorts and time points are presented in Table 3. At T46, CHOP-INTEND scores demonstrated a significant decline in cohorts B, C, D, and E, whereas cohort A exhibited an improvement. A minimum 4-point increase was observed across all cohorts, with cohort A showing the highest percentage of significant improvement at 93.5%. In cohort A, 21.7% of the patients in cohort B and 10.2% in cohort C 2% reached a maximum score of 64. Patients in cohort C who achieved 40 and 64 points, as well as the only patient in cohort D who achieved 40 points, were classified as type 1c.

**Table 3:** Comparison of treatment age groups based on CHOP-INTEND score.

CHOP-INTEND score	The age at the initiation of treatment					Total (*n* = 310)	*p*- value
	Cohort A (*n* = 46)	Cohort B (*n* = 147)	Cohort C (*n* = 49)	Cohort D (*n* = 35)	Cohort E (*n* = 33)		
Baseline	22.00 (15.00–29.25)^c^	19.00 (12.00–28.00)^c^	14.00 (7.00–22.00)^b^	9.00 (5.00–14.00)^ab^	6.00 (3.00–11.00)^a^	16.00 (8.00–24.25)	**<0.001**
T6	39.50 (27.00–45.00)^c^	33.50 (20.25–44.00)^c^	21.00 (13.75–32.00)^b^	14.00 (8.00–26.00)^ab^	11.00 (7.50–14.00)^a^	28.00 (15.00–41.75)	**<0.001**
T14	50.00 (36.75–58.00)^b^	45.00 (32.75–56.00)^b^	20.50 (11.00–42.25)^a^	15.50 (7.50–31.50)^a^	13.00 (9.00–17.00)^a^	35.00 (15.00–53.00)	**<0.001**
T26	55.00 (41.50–62.00)^c^	54.00 (31.50–60.00)^bc^	20.50 (7.50–48.75)^ab^	15.00 (5.00–23.00)^a^	12.00 (8.00–17.50)^a^	39.50 (12.75–58.00)	**<0.001**
T38	57.00 (47.50–64.00)^b^	60.00 (31.50–63.00)^b^	35.00 (13.50–58.75)^ab^	22.00 (6.00–31.00)^ab^	12.50 (8.25–18.50)^a^	31.50 (12.75–60.00)	**<0.001**
T46	60.00 (40.00–60.00)	43.00 (39.00–43.00)	18.00 (17.00–18.00)	15.50 (9.00–15.50)	10.00 (5.00–19.50)	22.00 (12.00–60.00)	0.068
Post-treatment	43.50 (32.00–60.00)^c^	39.00 (21.00–54.00)^c^	20.00 (12.50–32.00)^b^	15.00 (7.00–25.00)^ab^	11.00 (6.50–16.00)^a^	28.50 (14.00–45.00)	**<0.001**
4 points increase *n* (%)	43 (93.5)^a^	119 (81.0)^a^	35 (71.4)^ab^	19 (54.3)^bc^	11 (33.3)^c^	227 (73.2)	**<0.001**
40 points increase *n* (%)	31 (67.4)^a^	72 (49.0)^a^	10 (20.4)^b^	1 (2.9)^b^	0 (0.0)^b^	114 (36.8)	**<0.001**
64 points increase *n* (%)	10 (21.7)^a^	15 (10.2)^ab^	1 (2.0)^b^	0 (0.0)^b^	0 (0.0)^b^	26 (8.4)	**<0.001**

SMA, Spinal Muscular Atrophy. Statistically significant values are in bold. Values are shown as median (1st-3rd quartiles). In post hoc comparisons the same superscripts indicate similarities among groups, while different superscripts indicate a statistically significant differences among groups.

Univariate and multivariate logistic regression analyses for a 4-point increase in the CHOP-INTEND score post-treatment are presented in Table 4. Significant factors influencing a 4-point increase included baseline CHOP-INTEND score, spontaneous respiration prior to treatment, cohort C, cohort D, and cohort E patients who were tube-fed or underwent gastrostomy prior to treatment, and SMA type 1c. Supplementary Table 2 provides the results of linear regression analysis to identify the predictors of post-treatment CHOP-INTEND score improvement.

**Table 4:** Univariate and multiple logistic regression analysis to identify predictors of a 4-point increase in patients with and without the condition.

Variable	Univariate		Multiple	
	OR (95% CI)	*p*	OR (95% CI)	*p*
The age at the initiation of treatment				
Cohort A	1.00	–	–	–
Cohort B	0.297 (0.086–1.025)	0.055	–	–
Cohort C	0.174 (0.046–0.656)	**0.010**	–	–
Cohort D	0.083 (0.022–0.318)	**<0.001**	–	–
Cohort E	0.035 (0.009–0.138)	**<0.001**	–	–
Gender				
Female	1.00	–	–	–
Male	1.666 (0.997–2.782)	0.051	–	–
SMN copies				
2 copies	1.00	–	–	–
3 copies	0.383 (0.046–3.163)	0.383	–	–
Types of SMA				
Type 1a	1.00	-	–	–
Type 1b	1.480 (0.866–2.531)	0.152	–	–
Type 1c	3.538 (1.182–10.587)	**0.024**	–	–
Pre-treatment respiratory				
24-h	1.00	–	–	–
Spontaneous	7.000 (4.000–12.250)	**<0.001**	–	–
Pre-treatment feeding				
Oral	1.00	–	1.00	
Tube	0.132 (0.067–0.261)	**<0.001**	0.244 (0.117–0.505)	**<0.001**
Gastrostomy	0.070 (0.034–0.144)	**<0.001**	0.176 (0.080–0.391)	**<0.001**
CHOP-INTEND score, at baseline	1.148 (1.104–1.193)	**<0.001**	1.106 (1.061–1.153)	**<0.001**

OR, Odds ratio; CI, confidence intervals. Bold values indicate statistically significant (*p*-value < 0.05). Omnibus test chi-square test = 100.971, *p*-value < 0.001; Hosmer and Lemeshow test = 5.006, *p*-value = 0.757 and Nagelkerke *R*^2^ = 0.405.

### Need for ventilatory support and enteral feeding

**Figure 2:**
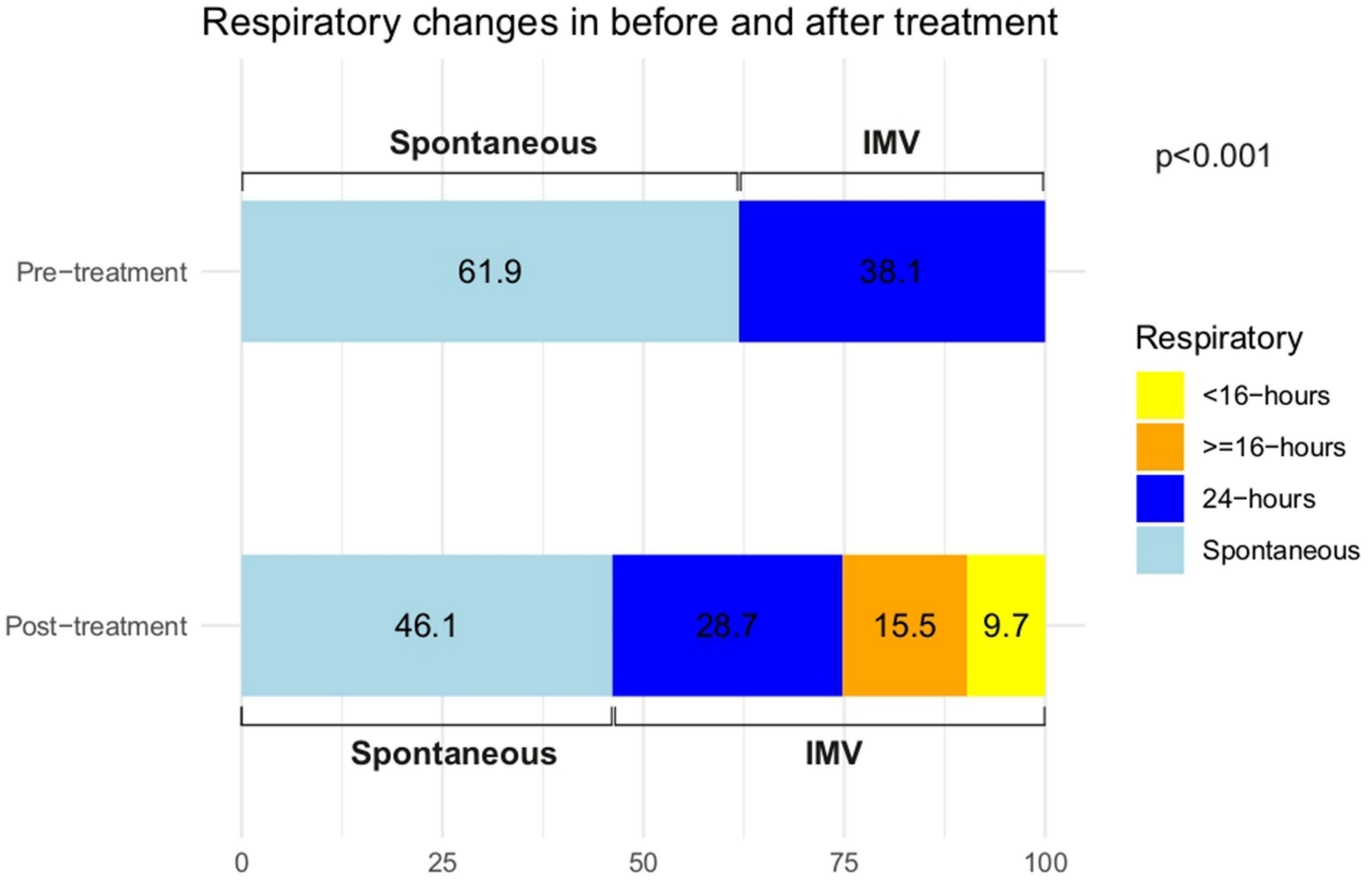
Respiratory changes before and after treatment. IMV, Invasive mechanical ventilation.

Pre-treatment, 38.1% of the patients required 24-h ventilatory support, while 61.9% had spontaneous respiration. Following treatment, the proportion of patients requiring ventilatory support increased to 53.9%. However, the percentage of patients requiring 24-h ventilatory support decreased by 28.7%, with 9.7% requiring <16 h and 15.5% requiring ≥16 h of support. The probability of requiring ventilatory support was significantly lower in cohort A than in the other cohorts (*p* < 0.0001; Figure 2; Supplementary Table 3). The post-hoc analysis of ventilator requirements by cohort is shown in Supplementary Table 4.

**Figure 3:**
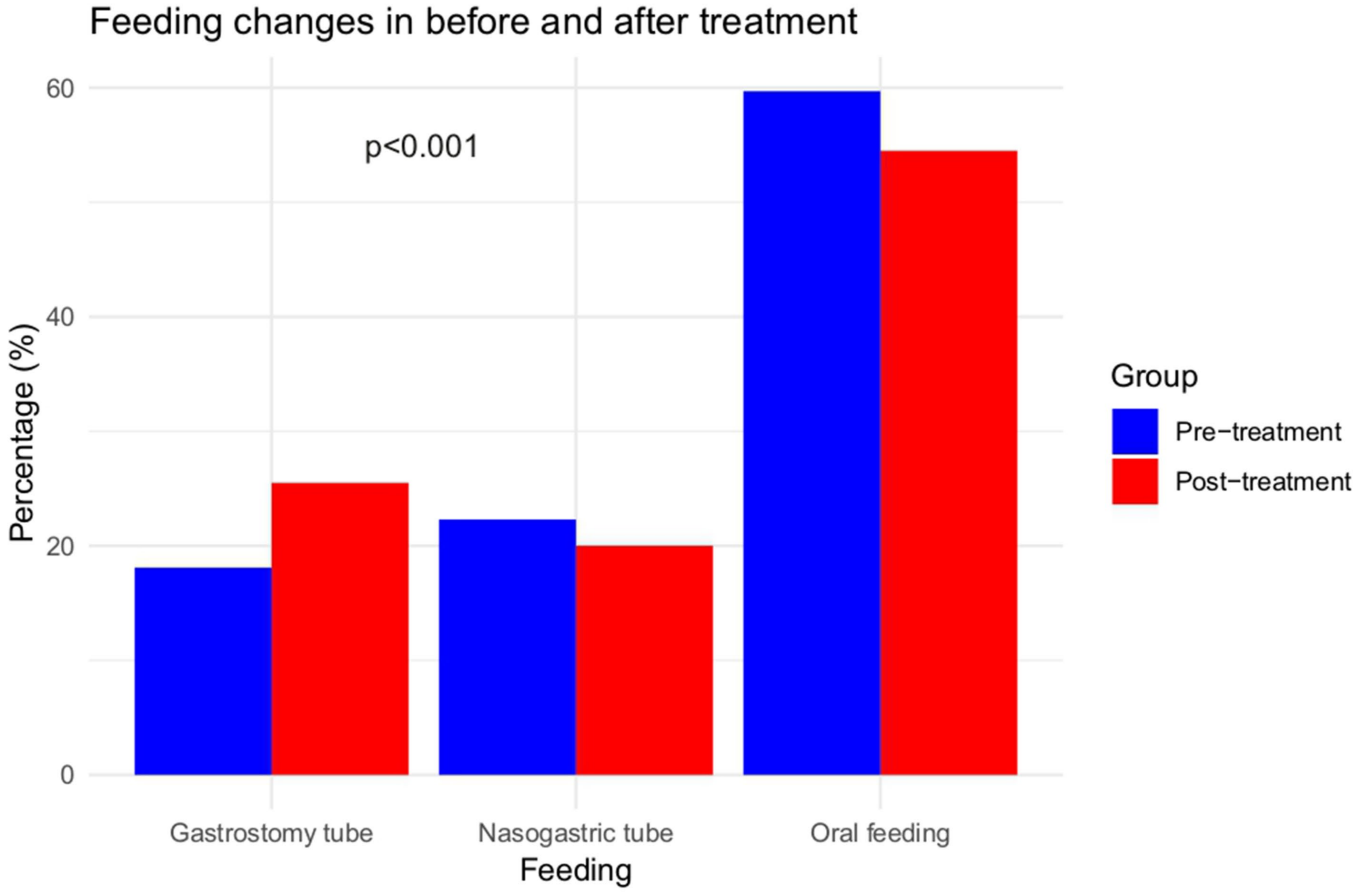
Feeding status before and after treatment.

Regarding feeding status, 59.7% of patients were orally fed, 22.3% were tube-fed, and 18.1% underwent gastrostomy before treatment. After treatment, 54.5% of the patients were orally fed, 20% were tube-fed, and 25.5% underwent a gastrostomy. Patients who started treatment before 3 months of age had a significantly lower need for tube feeding and gastrostomy than those in other cohorts (*p* < 0.0001; Figure 3; Supplementary Table 5). A post-hoc analysis of feeding status is presented in Supplementary Table 5.

### Adverse events

A total of 109 adverse events were recorded in 310 patients during nusinersen treatment, 46 of which were defined as serious (42.6%). Treatment was discontinued in only one patient who developed hydrocephalus. The most frequent adverse events were pneumonia (22%), proteinuria (17.4%), and acute respiratory failure (9.2) (Supplementary Table 6). The rate of occurrence of adverse events was <5%.

### Mortality

The mortality rate was 21.3% (n = 66), with 68.2% of the deaths occurring within the first year (Table 1). Survival probabilities are shown in Supplementary Figure S2. The causes of death are listed in Supplementary Table 7.

## Discussion

This prospective observational cohort study, conducted across 24 institutions in Turkey, provided real-world follow-up data on the clinical and treatment outcomes of SMA1 patients. To our knowledge, this is the first study to report a cohort of 310 SMA1 patients followed up for up to 4 years, with nusinersen treatment ranging from 4 to 15 cycles. Treatment was initiated in children as young as 0 days and up to 120 months old. A key feature of our cohort was the inclusion of presymptomatic and severely symptomatic patients in the study.

The evaluation of treatment outcomes across a broad age range may provide crucial insights into the optimal timing for initiating effective SMA interventions. In a 4-year follow-up of 48 SMA type 1 patients aged 0–12 years, including some on invasive mechanical ventilation (IMV), Pine et al. observed significant improvements in CHOP-INTEND scores among patients younger than 4 years (17). Similarly, Lusakowska et al. reported a CHOP-INTEND increase of ≥4 points in 50% of their SMA type 1 cohort by 14 months in a 30-month real-world study (18). Tachibana et al., in a follow-up exceeding 4 years, demonstrated that patients who began treatment before 13.1 weeks of age exhibited the most favorable outcomes (19). Additionally, the SMArtCARE study group showed the greatest improvements in CHOP-INTEND scores among patients who initiated treatment before 2 years of age (20).

Our study included severely symptomatic patients, primarily older children, on 24-h ventilatory support awaiting SMA treatment. This contributed to a higher mortality rate in the first 2 years compared to other studies. Therefore, CHOP-INTEND scores and motor milestones were lower in patients included in the first year. An increase in CHOP-INTEND scores was observed across all the cohorts. Notably, the first three cohorts, in which treatment was initiated before 12 months of age, demonstrated greater score improvement than those who began treatment after 12 months. Previous SMA-type 1 studies showed that clinically meaningful improvement was considered in CHOP-INTEND score ≥ 4, whereas the maximum benefit from treatment was accepted with a score of 40 or above (5, 17–19). In patients receiving ventilatory support, gastrostomy, tube feeding, and initiation of treatment after 6 months of age (cohorts C-E) were associated with a decreased probability of achieving a ≥ 4-point increase in the CHOP-INTEND scores. Conversely, the most favorable outcomes were observed in patients with spontaneous respiration (odds ratio [OR]: 7) and SMA type 1c (OR:3.5) (Table 4).

None of the patients who initiated treatment after 12 months achieved motor milestones as defined by the World Health Organization (WHO) (data not shown). The probability of achieving independent sitting was significantly higher in cohorts A and B, as well as in patients who commenced treatment before 12 months of age. However, independent sitting was observed in some patients up to 13 months of age. These threshold values may serve as critical indicators for evaluating the efficacy of treatment in patients with spinal muscular atrophy (SMA). Given that previous studies on patients with symptomatic SMA type 1 identified a maximum age of 14 months for optimal motor benefit (18, 20), it can be inferred that initiating treatment beyond 13–14 months may have limited efficacy in these patients. Therefore, this age range might be a crucial threshold for treatment initiation.

Randomized controlled trials and meta-analyses have established the relative safety profile of nusinersen (21, 22). In the EMBRACE and ENDEAR trials, no nusinersen-related adverse events (AEs) led to study termination (5, 22). According to a comprehensive meta-analysis, the most common AEs were fever (40%), upper respiratory tract infections (39.9%), and pneumonia (26.6%) (23). In our study, only one treatment discontinuation occurred because of AEs; one patient developed hydrocephalus following treatment, necessitating discontinuation. However, no other adverse effects, including serious adverse effects, required treatment cessation.

Mendonça et al. and Sansone et al. reported that nearly all patients receiving invasive mechanical ventilation (IMV) maintained stable respiratory support after treatment (24, 25). While Sansone et al. did not observe significant post-treatment changes in patients receiving non-invasive ventilation (NIV), the majority of NIV-dependent patients in the study by Mendonça et al. demonstrated a reduction in daily ventilation hours. Additionally, Sansone et al. noted improved ventilatory function in patients who initiated treatment before the age of two (24, 25). In the SMArtCARE study, 40% of the patients in the ≤2-year cohort and 84% of those in the >2-year cohort required ventilatory support at baseline (20). Notably, the need for ventilation has increased over time after treatment. In a long-term follow-up study of 303 patients with SMA types 1 and 2 in Japan, permanent ventilatory support was discontinued in two (0.7%) patients with SMA type 1 (19). Similarly, in an Italian cohort of 48 patients with SMA type 1, 13 required tracheostomy at the start of treatment. After 4 years of treatment, two of these patients were able to breathe spontaneously for 4–6 h per day (17). A comprehensive systematic review of 14 studies on nusinersen indicated that among 172 patients, only one (0.6%) was successfully weaned off mechanical ventilation (26).

In our study, the requirement for mechanical ventilation increased following treatment. However, when comparing cohort A with other cohorts, the probability of requiring ventilatory support at all times was significantly lower in cohort A. Additionally, while 38.1% of the study group required 24-h mechanical ventilation before treatment, this need was reduced in approximately 10% of the patients after treatment. This finding is consistent with the existing literature, indicating that nusinersen reduces the need for mechanical ventilation. Statistical analysis further revealed a significant reduction in mechanical ventilation hours in patients who initiated treatment before 3 months of age (Cohort A). Considering both our findings and the existing literature, these results suggest that the greatest efficacy of nusinersen in SMA patients requiring mechanical ventilation is achieved when treatment is initiated within the first 3 months, a period during which respiratory muscle involvement is not yet severe. Moreover, early nusinersen treatment may reverse mechanical ventilation dependency in these patients, particularly when initiated in cases where ventilatory support is required due to comorbid conditions, such as lower respiratory tract infections.

Previous studies have indicated that nusinersen is effective in improving motor function, but does not significantly affect bulbar function (27, 28). Van der Heul et al. focused on the study of bulbar function in SMA type 1 patients receiving nusinersen and found that swallowing ability declined between 8 and 12 months of age under treatment (29). Our findings corroborate these results, showing that nusinersen does not significantly improve bulbar function in symptomatic patients, particularly if treatment is not initiated during the presymptomatic stages. It is hypothesized that administering higher doses of nusinersen may enhance its effects on high cervical nuclei, potentially yielding better outcomes. The ongoing DEVOTE study, which evaluated the safety of high-dose nusinersen, may provide further insights, and its clinical results could help clarify this potential (30).

### Limitations

Our study has limitations as it was designed as a natural longitudinal data collection study with no control groups for comparison. Additionally, no neurophysiological exploratory examination was performed in our cohort. While evaluating the bulbar functions of the patients, evaluation of their speaking skills was not included in the study.

## Conclusion

The study involved 24 centers across Turkey, and all the participants were trained in the study. The cohort ranged from pre-symptomatic infants to children up to 120 months old. In conclusion, patients treated before 3 months of age demonstrated the most favorable responses in terms of motor outcomes, bulbar function, and ventilator dependency. Best motor responses were observed in patients treated at or before 13 months of age. However, no significant improvements in bulbar function or ventilatory support were observed in the symptomatic patients.

## Data Availability

The original contributions presented in the study are included in the article/Supplementary material, further inquiries can be directed to the corresponding author/s.

## Footnotes

^1^ www.r-project.org

## Author’s note

ÖB, DY, KBÇ and GD are members of the national SMA scientific board.

## Group member of Turkish SMA Study Group

Ömer Bektaş, Division of Pediatric Neurology, Department of Pediatrics, Faculty of Medicine, Ankara University, Ankara, Türkiye; Murat Gülşen, Ministry of Health, General Directorate of Health Services, Department of Autism, Mental Special Needs, and Rare Diseases, Ankara, Türkiye; Onur Burak Dursun, Ministry of Health, General Directorate of Health Services, Department of Autism, Mental Special Needs, and Rare Diseases, Ankara, Türkiye; Ahmet Tekin, Ministry of Health, Ankara, Türkiye; Deniz Yüksel, Division of Pediatric Neurology, Department of Pediatrics, Dr. Sami Ulus Research and Training Hospital, University of Health Sciences Turkey, Ankara, Türkiye; Ercan Demir, Division of Pediatric Neurology, Department of Pediatrics, Faculty of Medicine, Gazi University, Ankara, Türkiye; Gülten Öztürk, Division of Pediatric Neurology, Department of Pediatrics, Faculty of Medicine, Marmara University, İstanbul, Türkiye; Sema Saltık, Division of Pediatric Neurology, Department of Pediatrics, Faculty of Medicine, İstanbul University Cerrahpaşa, İstanbul, Türkiye; Özlem Hergüler, Division of Pediatric Neurology, Department of Pediatrics, Faculty of Medicine, Çukurova University, Adana, Türkiye; Ayşe Aysima Özçelik, Division of Pediatric Neurology, Department of Pediatrics, Faculty of Medicine, Gaziantep University, Gaziantep, Türkiye; Hüseyin Tan, Division of Pediatric Neurology, Department of Pediatrics, Faculty of Medicine, Atatürk University, Erzurum, Türkiye; Bilge Özgör, Division of Pediatric Neurology, Department of Pediatrics, Faculty of Medicine, Turgut Ozal Research Center, Inonu University, Malatya, Türkiye; Arzu Ekici, Division of Pediatric Neurology, Department of Pediatrics, Bursa Yüksek İhtisas Research and Training Hospital, University of Health Sciences Turkey, Ankara, Türkiye; Merve Feyza Yüksel, Division of Pediatric Neurology, Department of Pediatrics, Faculty of Medicine, Ankara University, Ankara, Türkiye; Süleyman Şahin, Division of Pediatric Neurology, Department of Pediatrics, Faculty of Medicine, Ankara University, Ankara, Türkiye; Özgür Duman, Division of Pediatric Neurology, Department of Pediatrics, Faculty of Medicine, Akadeniz University, Antalya, Türkiye; Mustafa Kömür, Division of Pediatric Neurology, Department of Pediatrics, Faculty of Medicine, Mersin University, Mersin, Türkiye; Figen Baydan, Division of Pediatric Neurology, Department of Pediatrics, Tepecik Research and Training Hospital, İzmir, Türkiye; Edibe Pembegül Yıldız, Division of Pediatric Neurology, Department of Pediatrics, İstanbul University, İstanbul, Türkiye; Bülent Kara, Division of Pediatric Neurology, Department of Pediatrics, Faculty of Medicine, Kocaeli University, Kocaeli, Türkiye; Uluç Yiş, Division of Pediatric Neurology, Department of Pediatrics, Faculty of Medicine, Dokuz Eylül University, İzmir, Türkiye; Seda Kanmaz, Division of Pediatric Neurology, Department of Pediatrics, Faculty of Medicine, Ege University, İzmir, Türkiye; Kürşat Bora Çarman, Division of Pediatric Neurology, Department of Pediatrics, Faculty of Medicine, Eskişehir Osmangazi University, Eskişehir, Türkiye; Elif Acar Arslan, Division of Pediatric Neurology, Department of Pediatrics, Faculty of Medicine, Karadeniz Teknik University, Trabzon, Türkiye; Mehmet Canpolat, Division of Pediatric Neurology, Department of Pediatrics, Faculty of Medicine, Erciyes University, Kayseri, Türkiye; Ahmet Sami Güven, Division of Pediatric Neurology, Department of Pediatrics, Faculty of Medicine, Necmettin Erbakan University, Konya, Türkiye; Gökçen Öztuncer, Division of Pediatric Neurology, Department of Pediatrics, Faculty of Medicine, Ondokuz Mayıs University, Samsun, Türkiye; Aycan Ünalp, Division of Pediatric Neurology, Department of Pediatrics, Faculty of Medicine, Dr. Behçet Uz Children’s Education and Research Hospital, University of Health Sciences Turkey Izmir, Izmir, Türkiye; Didem Ardıçlı, Division of Pediatric Neurology, Department of Pediatrics, Ankara City Hospital, University of Health Sciences Turkey, Ankara, Türkiye; Aynur Ayşe Karaduman, Department of Physiotherapy and Rehabilitation, Faculty of Medicine, Lokman Hekim University, Ankara, Türkiye; Gökmen Zararsız, Department of Biostatistics, Erciyes University School of Medicine, Kayseri, Türkiye; Nurşah Yeniay Süt, Division of Pediatric Neurology, Department of Pediatrics, Faculty of Medicine, Ankara University, Ankara, Türkiye; Olcay Ünver, Division of Pediatric Neurology, Department of Pediatrics, Faculty of Medicine, Marmara University, İstanbul, Türkiye; Tuğçe Damla Dilek, Division of Pediatric Neurology, Department of Pediatrics, Faculty of Medicine, İstanbul University Cerrahpaşa, İstanbul, Türkiye; Eif Yıldırım, Division of Pediatric Neurology, Department of Pediatrics, Faculty of Medicine, Atatürk University, Erzurum, Türkiye; Faruk İncecik, Division of Pediatric Neurology, Department of Pediatrics, Faculty of Medicine, Çukurova University, Adana, Türkiye; Meral Karadağ, Division of Pediatric Neurology, Department of Pediatrics, Faculty of Medicine, Turgut Ozal Research Center, Inonu University, Malatya, Türkiye; Mehmet Fatih Bütün, Division of Pediatric Neurology, Department of Pediatrics, Faculty of Medicine, Akadeniz University, Antalya, Türkiye; Burçin Gönüllü Polat, Division of Pediatric Neurology, Department of Pediatrics, Faculty of Medicine, Mersin University, Mersin, Türkiye; Yiğithan Güzin, Division of Pediatric Neurology, Department of Pediatrics, Tepecik Research and Training Hospital, İzmir, Türkiye; Mehmet Akif Kılıç, Division of Pediatric Neurology, Department of Pediatrics, İstanbul University, İstanbul, Türkiye; Adnan Deniz, Division of Pediatric Neurology, Department of Pediatrics, Faculty of Medicine, Kocaeli University, Kocaeli, Türkiye; Gamze Sarıkaya Uzan, Division of Pediatric Neurology, Department of Pediatrics, Faculty of Medicine, Dokuz Eylül University, İzmir, Türkiye; Sanem Yılmaz Keskin, Division of Pediatric Neurology, Department of Pediatrics, Faculty of Medicine, Ege University, İzmir, Türkiye; Fatih M. Özdemir, Division of Pediatric Neurology, Department of Pediatrics, Dr. Sami Ulus Research and Training Hospital, University of Health Sciences Turkey, Ankara, Türkiye; Coşkun Yarar, Division of Pediatric Neurology, Department of Pediatrics, Faculty of Medicine, Eskişehir Osmangazi University, Eskişehir, Türkiye; Nihal Yıldız, Division of Pediatric Neurology, Department of Pediatrics, Faculty of Medicine, Karadeniz Teknik University, Trabzon, Türkiye; Hamit Acer, Division of Pediatric Neurology, Department of Pediatrics, Faculty of Medicine, Erciyes University, Kayseri, Türkiye; Hüseyin Çaksen, Division of Pediatric Neurology, Department of Pediatrics, Faculty of Medicine, Necmettin Erbakan University, Konya, Türkiye; Seren Aydın, Division of Pediatric Neurology, Department of Pediatrics, Faculty of Medicine, Ondokuz Mayıs University, Samsun, Türkiye; Pakize Karaoğlu, Division of Pediatric Neurology, Department of Pediatrics, Faculty of Medicine, Dr. Behçet Uz Children’s Education and Research Hospital, University of Health Sciences Turkey Izmir, Izmir, Türkiye; Ayşe Neşe Çıtak, Division of Pediatric Neurology, Department of Pediatrics, Ankara City Hospital, University of Health Sciences Turkey, Ankara, Türkiye; Tanıl Kendirli, Turkish Medicines and Medical Devices Agency, Ankara, Türkiye; Birkan Sonel Tur, Department of Physical Therapy and Rehabilitation, Faculty of Medicine, Ankara University, Ankara, Türkiye; Gülcan Akyüz, Division of Pediatric Neurology, Department of Pediatrics, Faculty of Medicine, Marmara University, İstanbul, Türkiye; Ceren Bibinoğlu Amirov, Division of Pediatric Neurology, Department of Pediatrics, Faculty of Medicine, İstanbul University Cerrahpaşa, İstanbul, Türkiye; Gülen Gül Mert, Division of Pediatric Neurology, Department of Pediatrics, Faculty of Medicine, Çukurova University, Adana, Türkiye; Mahmut Aslan, Division of Pediatric Neurology, Department of Pediatrics, Faculty of Medicine, Turgut Ozal Research Center, Inonu University, Malatya, Türkiye; Şenay Haspolat, Division of Pediatric Neurology, Department of Pediatrics, Faculty of Medicine, Akadeniz University, Antalya, Türkiye; Özlem Ersoy, Division of Pediatric Neurology, Department of Pediatrics, Faculty of Medicine, Mersin University, Mersin, Türkiye; Osman Kipoğlu, Division of Pediatric Neurology, Department of Pediatrics, İstanbul University, İstanbul, Türkiye; Defne Alikılıç, Division of Pediatric Neurology, Department of Pediatrics, Faculty of Medicine, Kocaeli University, Kocaeli, Türkiye; Hasan Tekgül, Division of Pediatric Neurology, Department of Pediatrics, Faculty of Medicine, Ege University, İzmir, Türkiye; Ülkühan Kaya, Division of Pediatric Neurology, Department of Pediatrics, Dr. Sami Ulus Research and Training Hospital, University of Health Sciences Turkey, Ankara, Türkiye; Arife Derda Yücel Şen, Division of Pediatric Neurology, Department of Pediatrics, Faculty of Medicine, Eskişehir Osmangazi University, Eskişehir, Türkiye; Pınar Özkan Kart, Division of Pediatric Neurology, Department of Pediatrics, Faculty of Medicine, Karadeniz Teknik University, Trabzon, Türkiye; Hüseyin Per, Division of Pediatric Neurology, Department of Pediatrics, Faculty of Medicine, Erciyes University, Kayseri, Türkiye; Ayşe Aksoy, Division of Pediatric Neurology, Department of Pediatrics, Faculty of Medicine, Ondokuz Mayıs University, Samsun, Türkiye; Ünsal Yılmaz, Division of Pediatric Neurology, Department of Pediatrics, Faculty of Medicine, Dr. Behçet Uz Children’s Education and Research Hospital, University of Health Sciences Turkey Izmir, Izmir, Türkiye; Serap Teber, Division of Pediatric Neurology, Department of Pediatrics, Faculty of Medicine, Ankara University, Ankara, Türkiye; Özben Akıncı Göktaş, Division of Pediatric Neurology, Department of Pediatrics, Faculty of Medicine, Ankara University, Ankara, Türkiye; Dilşad Türkdoğan, Division of Pediatric Neurology, Department of Pediatrics, Faculty of Medicine, Marmara University, İstanbul, Türkiye; Fitnat Uluğ, Division of Pediatric Neurology, Department of Pediatrics, Faculty of Medicine, İstanbul University Cerrahpaşa, İstanbul, Türkiye; Serap Bilge, Division of Pediatric Neurology, Department of Pediatrics, Faculty of Medicine, Çukurova University, Adana, Türkiye; Orhan Coşkun, Division of Pediatric Neurology, Department of Pediatrics, İstanbul University, İstanbul, Türkiye; Merve Öztürk, Division of Pediatric Neurology, Department of Pediatrics, Faculty of Medicine, Kocaeli University, Kocaeli, Türkiye; Esra Atmaca, Division of Pediatric Neurology, Department of Pediatrics, Dr. Sami Ulus Research and Training Hospital, University of Health Sciences Turkey, Ankara, Türkiye; Ali Cansu, Division of Pediatric Neurology, Department of Pediatrics, Faculty of Medicine, Karadeniz Teknik University, Trabzon, Türkiye; Hakan Gümüş, Division of Pediatric Neurology, Department of Pediatrics, Faculty of Medicine, Erciyes University, Kayseri, Türkiye; Evrim Karadağ Saygı, Department of Physical Therapy and Rehabilitation, Faculty of Medicine, Marmara University, İstanbul, Türkiye; Ömer Karaca, Division of Pediatric Neurology, Department of Pediatrics, Faculty of Medicine, Kocaeli University, Kocaeli, Türkiye; Serdal Güngör, Division of Pediatric Neurology, Department of Pediatrics, Faculty of Medicine, Turgut Ozal Research Center, Inonu University, Malatya, Türkiye; Nazan Çobanoğlu, Division of Pediatric Pulmonology, Department of Pediatrics, Faculty of Medicine, Ankara University, Ankara, Türkiye; Melek Sezgin, Department of Physical Therapy and Rehabilitation, Faculty of Medicine, Mersin University, Mersin, Türkiye; Miraç Yıldırım, Division of Pediatric Neurology, Department of Pediatrics, Faculty of Medicine, Ankara University, Ankara, Türkiye; Yasemin Gökdemir, Division of Pediatric Pulmonology, Department of Pediatrics, Faculty of Medicine, Marmara University, İstanbul, Türkiye; Esma Şengenç, Division of Pediatric Neurology, Department of Pediatrics, Faculty of Medicine, İstanbul University Cerrahpaşa, İstanbul, Türkiye; Yılmaz Zindar, Division of Pediatric Neurology, Department of Pediatrics, Faculty of Medicine, İstanbul University Cerrahpaşa, İstanbul, Türkiye; Özlem Yayıcı Köken, Division of Pediatric Neurology, Department of Pediatrics, Faculty of Medicine, Akadeniz University, Antalya, Türkiye; Mesut Güngör, Division of Pediatric Neurology, Department of Pediatrics, Faculty of Medicine, Selçuk University, Konya, Türkiye; Serkan Kırık, Division of Pediatric Neurology, Department of Pediatrics, Elazığ Fethi Sekin City Hospital, Health Sciences University, Elazığ, Türkiye; Meltem Çobanoğulları Direk, Division of Pediatric Neurology, Department of Pediatrics, Faculty of Medicine, Mersin University, Mersin, Türkiye; Gülhis Deda, Turkish Medicines and Medical Devices Agency, Ankara, Türkiye.

## Supplementary material

Supplementary Figure S1 The probability of achieving supported sitting across all cohorts.

Supplementary Figure S2 Survival probabilities.

## References

[1] Messina S Sframeli M. New treatments in spinal muscular atrophy: positive results and new challenges. J Clin Med. (2020) 9:2222. doi: 10.3390/jcm9072222, PMID: 32668756 PMC7408870

[2] Ceylan AC Erdem HB Şahin İ Agarwal M. SMN1 gene copy number analysis for spinal muscular atrophy (SMA) in a Turkish cohort by CODE-SEQ technology, an integrated solution for detection of SMN1 and SMN2 copy numbers and the "2+0" genotype. Neurol Sci. (2020) 41:2575–84. doi: 10.1007/s10072-020-04365-x, PMID: 32249332

[3] Hua Y Sahashi K Hung G Rigo F Passini MA Bennett CF. Antisense correction of SMN2 splicing in the CNS rescues necrosis in a type III SMA mouse model. Genes Dev. (2010) 24:1634–44. doi: 10.1101/gad.1941310, PMID: 20624852 PMC2912561

[4] Ogino S Gao S Leonard DG Paessler M Wilson RB. Inverse correlation between SMN1 and SMN2 copy numbers: evidence for gene conversion from SMN2 to SMN1. Eur J Hum Genet. (2003) 11:275–7. doi: 10.1038/sj.ejhg.5200957, PMID: 12673282

[5] Finkel RS Mercuri E Darras BT Connolly AM Kuntz NL Kirschner J. Nusinersen versus sham control in infantile-onset spinal muscular atrophy. N Engl J Med. (2017) 377:1723–32. doi: 10.1056/NEJMoa1702752, PMID: 29091570

[6] Crawford TO Swoboda KJ De Vivo DC Bertini E Hwu WL Finkel RS. Continued benefit of nusinersen initiated in the presymptomatic stage of spinal muscular atrophy: 5-year update of the NURTURE study. Muscle Nerve. (2023) 68:157–70. doi: 10.1002/mus.27853, PMID: 37409780

[7] Mercuri E Darras BT Chiriboga CA Day JW Campbell C Connolly AM. Nusinersen versus sham control in later-onset spinal muscular atrophy. N Engl J Med. (2018) 378:625–35. doi: 10.1056/NEJMoa1710504, PMID: 29443664

[8] Dangouloff T Vrščaj E Servais L Osredkar D SMA NBS World Study Group. Newborn screening programs for spinal muscular atrophy worldwide: where we stand and where to go. Neuromuscul Disord. (2021) 31:574–82. doi: 10.1016/j.nmd.2021.03.007, PMID: 33985857

[9] Gallagher GW Nowacek D Gutgsell O Callaghan BC. Comparison of the United Kingdom and United States approaches to approval of new neuromuscular therapies. Muscle Nerve. (2021) 64:641–50. doi: 10.1002/mus.27380, PMID: 34448221

[10] Wadman RI van der Pol WL Bosboom WM Asselman FL van den Berg LH Iannaccone ST. Drug treatment for spinal muscular atrophy type I. Cochrane Database Syst Rev. (2019) 12:CD006281. doi: 10.1002/14651858.CD006281.pub5, PMID: 31825542 PMC6905354

[11] Scheijmans FEV Cuppen I van Eijk RPA Wijngaarde CA Schoenmakers MAGC van der Woude DR. Population-based assessment of nusinersen efficacy in children with spinal muscular atrophy: a 3-year follow-up study. Brain Commun. (2022) 4:fcac269. doi: 10.1093/braincomms/fcac269, PMID: 36382221 PMC9651026

[12] Mercuri E Lucibello S Perulli M Coratti G de Sanctis R Pera MC. Longitudinal natural history of type I spinal muscular atrophy: a critical review. Orphanet J Rare Dis. (2020) 15:84. doi: 10.1186/s13023-020-01356-1, PMID: 32248834 PMC7132885

[13] Pane M Coratti G Sansone VA Messina S Catteruccia M Bruno C. Type I SMA “new natural history”: long-term data in nusinersen-treated patients. Ann Clin Transl Neurol. (2021) 8:548–57. doi: 10.1002/acn3.51276, PMID: 33547876 PMC7951096

[14] Audic F de la Banda MGG Bernoux D Ramirez-Garcia P Durigneux J Barnerias C. Effects of nusinersen after one year of treatment in 123 children with SMA type 1 or 2: a French real-life observational study. Orphanet J Rare Dis. (2020) 15:148. doi: 10.1186/s13023-020-01414-8, PMID: 32532349 PMC7291731

[15] Szabó L Gergely A Jakus R Fogarasi A Grosz Z Molnár MJ. Efficacy of nusinersen in type 1, 2 and 3 spinal muscular atrophy: real world data from Hungarian patients. Eur J Paediatr Neurol. (2020) 27:37–42. doi: 10.1016/j.ejpn.2020.05.002, PMID: 32456992

[16] Gülşen M Ceylan AC Bahsi T Çubukçu HC Dursun OB. Validation of SMA screening kits with SMN1 gene analysis in a Turkish cohort. Clin Chim Acta. (2024) 555:117793. doi: 10.1016/j.cca.2024.117793 38309554

[17] Pane M Coratti G Sansone VA Messina S Catteruccia M Bruno C. Type I spinal muscular atrophy patients treated with nusinersen: 4-year follow-up of motor, respiratory and bulbar function. Eur J Neurol. (2023) 30:1755–63. doi: 10.1111/ene.15768, PMID: 36880698

[18] Łusakowska A Wójcik A Frączek A Aragon-Gawińska K Potulska-Chromik A Baranowski P. Long-term nusinersen treatment across a wide spectrum of spinal muscular atrophy severity: a real-world experience. Orphanet J Rare Dis. (2023) 18:230. doi: 10.1186/s13023-023-02769-4, PMID: 37542300 PMC10401775

[19] Tachibana Y Takasaki S Hoshino M Makioka H Jin M. Real-world safety and effectiveness of nusinersen, a treatment for spinal muscular atrophy, in 401 Japanese patients: results from an interim analysis of post-marketing surveillance. Int J Neurosci. (2024) 134:153–62. doi: 10.1080/00207454.2022.2095270, PMID: 35787224

[20] Pechmann A Behrens M Dörnbrack K Tassoni A Stein S Vogt S. Effect of nusinersen on motor, respiratory and bulbar function in early-onset spinal muscular atrophy. Brain. (2023) 146:668–77. doi: 10.1093/brain/awac252, PMID: 35857854

[21] Abbas KS Eltaras MM El-Shahat NA Abdelazeem B Shaqfeh M Brašić JR. The safety and efficacy of Nusinersen in the treatment of spinal muscular atrophy: a systematic review and Meta-analysis of randomized controlled trials. Medicina (Kaunas). (2022) 58:213. doi: 10.3390/medicina58020213, PMID: 35208537 PMC8874456

[22] Acsadi G Crawford TO Müller-Felber W Shieh PB Richardson R Natarajan N. Safety and efficacy of nusinersen in spinal muscular atrophy: the EMBRACE study. Muscle Nerve. (2021) 63:668–77. doi: 10.1002/mus.27187, PMID: 33501671 PMC8248061

[23] Zhong ZJ Zheng PM Dou HH Wang JG. Adverse events in the treatment of spinal muscular atrophy in children and adolescents with nusinersen: a systematic review and meta-analysis. Front Pediatr. (2023) 11:1152318. doi: 10.3389/fped.2023.1152318, PMID: 37181426 PMC10167028

[24] De Holanda MR Jorge Polido G Ciro M Jorge Fontoura Solla D Conti Reed U Zanoteli E. Clinical outcomes in patients with spinal muscular atrophy type 1 treated with Nusinersen. J Neuromuscul Dis. (2021) 8:217–24. doi: 10.3233/JND-200533, PMID: 33459657

[25] Sansone VA Pirola A Albamonte E Pane M Lizio A D'Amico A. Respiratory needs in patients with type 1 spinal muscular atrophy treated with Nusinersen. J Pediatr. (2020) 219:223–228.e4. doi: 10.1016/j.jpeds.2019.12.047, PMID: 32035635

[26] Panagiotou P Kanaka-Gantenbein C Kaditis AG. Changes in Ventilatory support requirements of spinal muscular atrophy (SMA) patients post gene-based therapies. Children (Basel). (2022) 9:1207. doi: 10.3390/children9081207, PMID: 36010097 PMC9406975

[27] Weststrate H Stimpson G Thomas L Scoto M Johnson E Stewart A. Evolution of bulbar function in spinal muscular atrophy type 1 treated with nusinersen. Dev Med Child Neurol. (2022) 64:907–14. doi: 10.1111/dmcn.15171, PMID: 35103306 PMC9306995

[28] Pane M Stanca G Coratti G D’Amico A Sansone VA Berti B. Prognostic factors for tube feeding in type I SMA patients treated with disease-modifying therapies: a cohort study. Eur J Pediatr. (2024) 183:4735–45. doi: 10.1007/s00431-024-05735-9, PMID: 39210071 PMC11473555

[29] van der Heul AMB Cuppen I Wadman RI Asselman F Schoenmakers MAGC van de Woude DR. Feeding and swallowing problems in infants with spinal muscular atrophy type 1: an observational study. J Neuromuscul Dis. (2020) 7:323–30. doi: 10.3233/JND-190465, PMID: 32333596

[30] Finkel RS Day JW Pascual Pascual SI Ryan MM Mercuri E de Vivo DC. DEVOTE study exploring higher dose of Nusinersen in spinal muscular atrophy: study design and part a results. J Neuromuscul Dis. (2023) 10:813–23. doi: 10.3233/JND-221667, PMID: 37393513 PMC10578235

