# Supplementary figures and images for: Nusinersen for children with type I spinal muscular atrophy: 4 years’ clinical experience in Turkish cohort
Source: Front Neurol. 2025 Mar 27;16:1541507. doi: 10.3389/fneur.2025.1541507 (PMC11983886; doi:10.3389/fneur.2025.1541507)

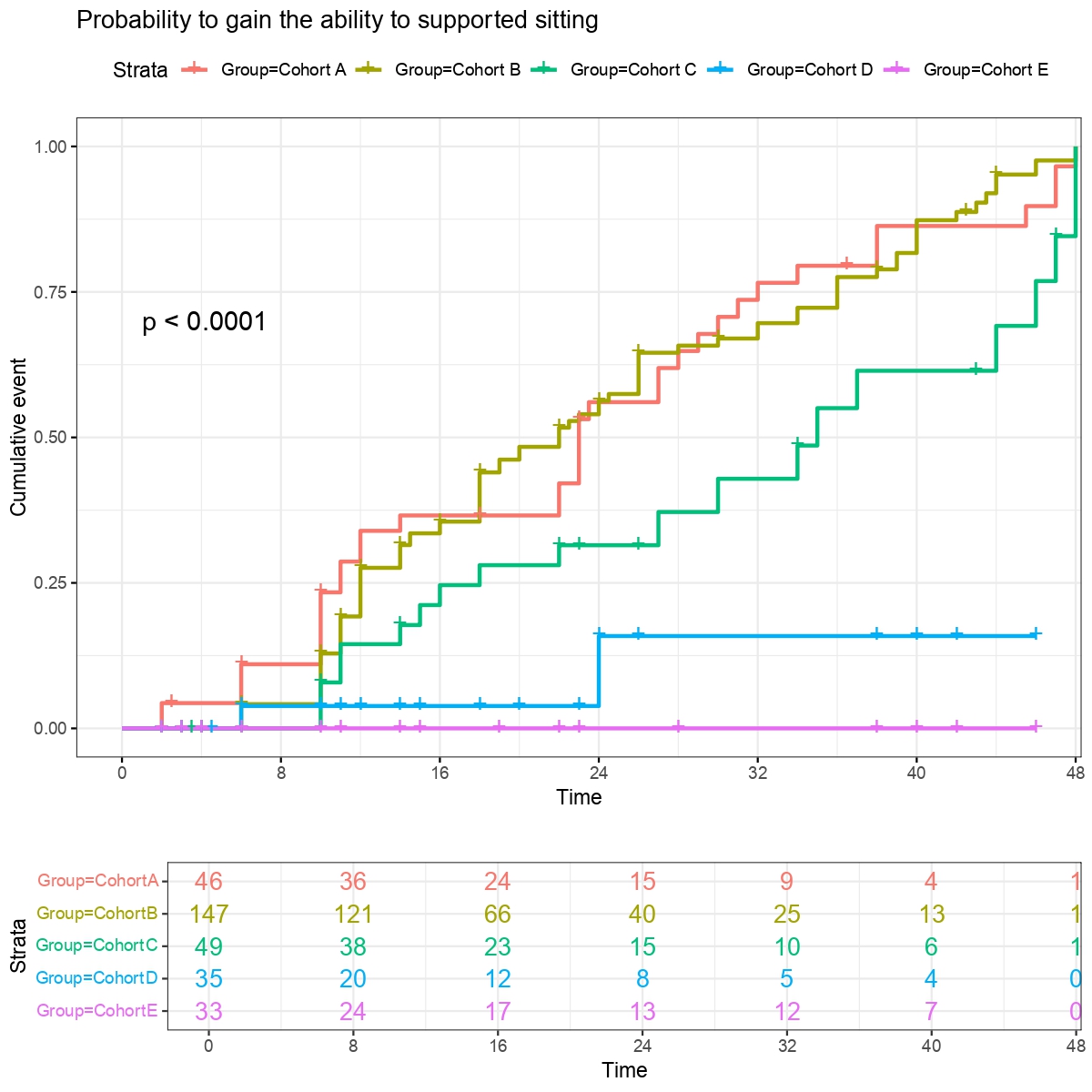

Supplement: Supplementary Figure S1 — The probability of achieving supported sitting across all cohorts. [file Image_1.JPEG]

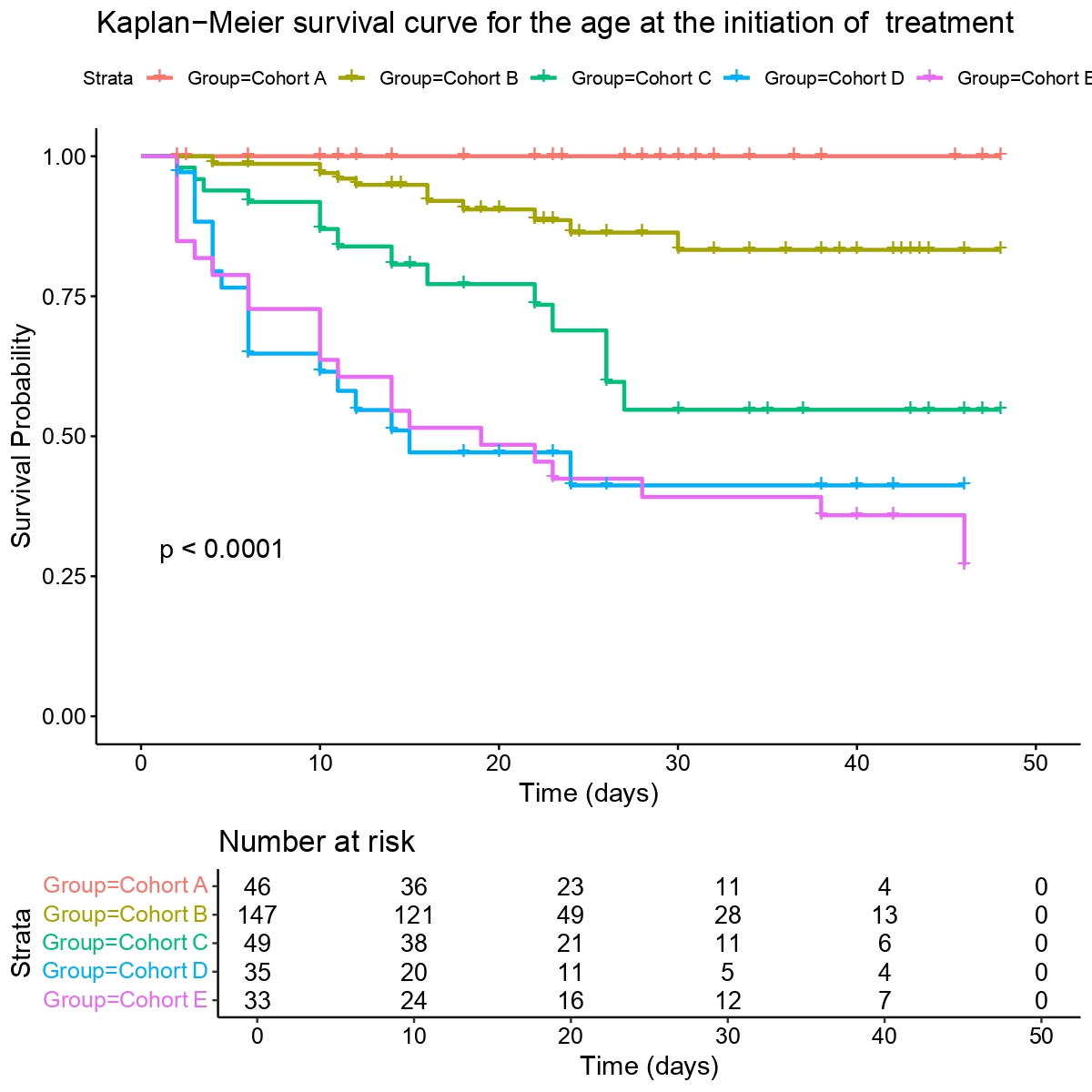

Supplement: Supplementary Figure S2 — Survival probabilities. [file Image_2.JPEG]
