# Supplementary material for: Nusinersen for children with type I spinal muscular atrophy: 4 years’ clinical experience in Turkish cohort
Source: Front Neurol. 2025 Mar 27;16:1541507. doi: 10.3389/fneur.2025.1541507 (PMC11983886; doi:10.3389/fneur.2025.1541507)
Supplement: Supplementary file 3 [file Table_1.DOCX]

**Supplementary Table 1.** Patient characteristics among the treatment age groups

| **Variable** | **The age at the initiation of treatment** | | | | | **Total**  **(*n*=310)** | ***p-value*** |
| --- | --- | --- | --- | --- | --- | --- | --- |
|  | **Cohort A**  **(*n*=46)** | **Cohort B**  **(*n*=147)** | **Cohort C**  **(*n*=49)** | **Cohort D**  **(*n*=35)** | **Cohort E**  **(*n*=33)** |  |  |
| **Gender (Female)** | 17 (37.0) | 78 (53.1) | 24 (49.0) | 14 (40.0) | 15 (45.5) | 148 (47.7) | 0.314 |
| **Age (months)** | 17.50 (9.75-34.25)^ad^ | 16.00 (10.00-29.00)^a^ | 13.00 (12.00-20.00)^a^ | 24.00 (18.00-30.00)^bd^ | 62.00 (46.00-83.50)^c^ | 18.25 (12.00-35.25) | **<0.001** |
| **Types of SMA** |  |  |  |  |  |  |  |
| Type 1a | 34 (73.9)^a^ | 77 (52.4)^ab^ | 18 (36.7)^b^ | 11 (31.4)^b^ | 16 (48.5)^ab^ | 156 (50.3) | **0.003** |
| Type 1b | 11 (23.9)^a^ | 53 (36.1)^ab^ | 26 (53.1)^b^ | 16 (45.7)^ab^ | 14 (42.4)^ab^ | 120 (38.7) |  |
| Type 1c | 1 (2.2)^a^ | 17 (11.6)^ab^ | 5 (10.2)^ab^ | 8 (22.9)^b^ | 3 (9.1)^ab^ | 34 (11.0) |  |
| **SMN copies (3 copies)** | 44 (95.7) | 142 (96.6) | 49 (100.0) | 35 (100.0) | 32 (97.0) | 302 (97.4) | 0.522 |
| **Exitus** | 0 (0.0)^a^ | 12 (8.2)^a^ | 14 (28.6)^b^ | 18 (51.4)^bc^ | 22 (66.7)^c^ | 66 (21.3) | **<0.001** |
| **Age at diagnosis (months)** | 2.00 (1.88-3.00)^a^ | 4.00 (3.00-5.00)^b^ | 5.00 (4.00-6.00)^d^ | 5.00 (4.00-5.01)^cd^ | 5.00 (3.50-5.01)^bd^ | 4.00 (3.00-5.00) | **<0.001** |
| **Age at onset of treatment (months)** | 3.00 (2.50-3.01)^c^ | 5.00 (4.00-5.01)^d^ | 7.00 (6.99-8.00)^a^ | 13.00 (12.00-14.50)^ab^ | 48.00 (36.00-75.00)^b^ | 5.00 (4.00-9.00) | **<0.001** |
| **Age at onset of SMA (months)** | 2.00 (1.00-2.50)^a^ | 2.00 (1.99-3.00)^b^ | 3.00 (2.00-4.00)^b^ | 3.00 (2.00-4.00)^b^ | 3.00 (2.00-3.01)^b^ | 2.50 (2.00-3.00) | **<0.001** |
| **Follow-up period (months)** | 20.00 (10.00-29.25) | 14.00 (10.00-24.50) | 14.00 (10.00-26.50) | 11.00 (4.50-23.00) | 19.00 (6.00-38.00) | 14.00 (10.00-26.00) | 0.296 |
| **Treatment delay** | 0.00 (0.00-1.00)^a^ | 1.00 (0.00-2.00)^c^ | 3.00 (1.75-4.00)^d^ | 10.00 (8.00-13.00)^b^ | 43.00 (30.50-70.00)^b^ | 1.50 (0.50-4.00) | **<0.001** |
| **The number of cyclus** | 7.00 (5.00-10.00)^a^ | 6.00 (4.00-8.00)^ab^ | 5.00 (4.00-5.50)^b^ | 4.00 (3.99-7.00)^b^ | 5.00 (4.00-6.50)^b^ | 5.00 (4.00-8.00) | **<0.001** |
| **Dose** | 7.00 (5.00-10.25)^a^ | 6.00 (4.00-9.00)^ac^ | 5.00 (4.00-5.50)^b^ | 4.00 (3.99-7.00)^bc^ | 5.00 (4.00-6.50)^bc^ | 5.00 (4.00-8.00) | **<0.001** |

**SMA:** Spinal Muscular Atrophy, **PrT:** Pre-treatment, **PoT:** Post-treatment. Statistically significant values ​​are in bold. Values ​​are shown as n (%) or median (1^st^-3^rd^ quartiles). In post hoc comparisons the same superscripts indicate similarities among groups, while different superscripts indicate a statistically significant differences among groups.
