# Supplementary material for: Nusinersen for children with type I spinal muscular atrophy: 4 years’ clinical experience in Turkish cohort
Source: Front Neurol. 2025 Mar 27;16:1541507. doi: 10.3389/fneur.2025.1541507 (PMC11983886; doi:10.3389/fneur.2025.1541507)
Supplement: Supplementary file 4 [file Table_2.DOCX]

**Supplementary Table 2. Linear regression analysis to identify predictors of a CHOP-INTEND score, at post-treatment.**

| **Variable** | **Univariate** | | **Multiple** | |
| --- | --- | --- | --- | --- |
|  | **Beta (95%CI)** | ***p*** | **Beta (95%CI)** | ***p*** |
| The age at the initiation of treatment | -8.587 (-10.151-(-7.024)) | **<0.001** | -3.039 (-4.414-(-1.664)) | **<0.001** |
| Gender | 6.522 (2.222-10.823) | **0.003** | - | **-** |
| SMN copies | -9.009 (-22.714-4.695) | 0.197 | - | **-** |
| Types of SMA | 7.673 (4.571-10.776) | **<0.001** | 4.151 (2.246-6.056) | **<0.001** |
| Pre-treatment respiratory | 7.622 (6.394-8.850) | **<0.001** | 2.127 (1.037-3.217) | **<0.001** |
| Pre-treatment feeding | -14.374 (-16.667- (-12.081)) | **<0.001** | - | **-** |
| CHOP-INTEND score, at baseline | 1.333 (1.220-1.446) | **<0.001** | 1.013 (0.889-1.137) | **<0.001** |

**CI:** confidence intervals. Bold values indicate statistically significant (*p-value*<0.05). R^2^=0.710.
