# Supplementary material for: Nusinersen for children with type I spinal muscular atrophy: 4 years’ clinical experience in Turkish cohort
Source: Front Neurol. 2025 Mar 27;16:1541507. doi: 10.3389/fneur.2025.1541507 (PMC11983886; doi:10.3389/fneur.2025.1541507)
Supplement: Supplementary file 5 [file Table_3.DOCX]

**Supplementary Table 3. Comparison of treatment age groups based on ventilator requirements**

| **Variable** | **The age at the initiation of treatment** | | | | | **Total**  **(*n*=310)** | ***p-value*** |
| --- | --- | --- | --- | --- | --- | --- | --- |
|  | **Cohort A**  **(*n*=46)** | **Cohort B**  **(*n*=147)** | **Cohort C**  **(*n*=49)** | **Cohort D**  **(*n*=35)** | **Cohort E**  **(*n*=33)** |  |  |
| **Pre-treatment respiratory** |  |  |  |  |  |  |  |
| 24-hours | 5 (10.9)^a^ | 26 (17.7)^a^ | 25 (51.0)^b^ | 29 (82.9)^c^ | 33 (100.0)^c^ | 118 (38.1) | **<0.001** |
| Spontaneous | 41 (89.1)^a^ | 121 (82.3)^a^ | 24 (49.0)^b^ | 6 (17.1)^c^ | 0 (0.0)^c^ | 192 (61.9) |  |
| **Post-treatment respiratory** |  |  |  |  |  |  |  |
| 24-hours | 1 (2.2)^a^ | 19 (12.9)^a^ | 24 (49.0)^b^ | 20 (57.1)^b^ | 25 (75.8)^b^ | 89 (28.7) | **<0.001** |
| >=16-hours | 2 (4.3)^a^ | 22 (15.0)^a^ | 8 (16.3)^a^ | 8 (22.9)^a^ | 8 (24.2)^a^ | 48 (15.5) |  |
| <16-hours | 2 (4.3)^a^ | 19 (12.9)^a^ | 4 (8.2)^a^ | 5 (14.3)^a^ | 0 (0.0)^a^ | 30 (9.7) |  |
| Spontaneous | 41 (89.1)^a^ | 87 (59.2)^b^ | 13 (26.5)^c^ | 2 (5.7)^cd^ | 0 (0.0)^d^ | 143 (46.1) |  |

**SMA:** Spinal Muscular Atrophy. Statistically significant values ​​are in bold. Values ​​are shown as *n* (%). In post hoc comparisons the same letters show similarities among groups, different letters show differences among groups.
