# Supplementary material for: Nusinersen for children with type I spinal muscular atrophy: 4 years’ clinical experience in Turkish cohort
Source: Front Neurol. 2025 Mar 27;16:1541507. doi: 10.3389/fneur.2025.1541507 (PMC11983886; doi:10.3389/fneur.2025.1541507)
Supplement: Supplementary file 6 [file Table_4.DOCX]

**Table 4. Univariate and multiple logistic regression analysis to identify predictors of a 4-point increase in patients with and without the condition**

| **Variable** | **Univariate** | | **Multiple** | |
| --- | --- | --- | --- | --- |
|  | **OR (95%CI)** | ***p*** | **OR (95%CI)** | ***p*** |
| **The age at the initiation of treatment** |  |  |  |  |
| Cohort A | 1.00 | - | - | - |
| Cohort B | 0.297 (0.086-1.025) | 0.055 | - | - |
| Cohort C | 0.174 (0.046-0.656) | **0.010** | - | - |
| Cohort D | 0.083 (0.022-0.318) | **<0.001** | - | - |
| Cohort E | 0.035 (0.009-0.138) | **<0.001** | - | - |
| **Gender** |  |  |  |  |
| Female | 1.00 | - | - | - |
| Male | 1.666 (0.997-2.782) | 0.051 | - | - |
| **SMN copies** |  |  |  |  |
| 2 copies | 1.00 | - | - | - |
| 3 copies | 0.383 (0.046-3.163) | 0.383 | - | - |
| **Types of SMA** |  |  |  |  |
| Type 1a | 1.00 | - | - | - |
| Type 1b | 1.480 (0.866-2.531) | 0.152 | - | - |
| Type 1c | 3.538 (1.182-10.587) | **0.024** | - | - |
| **Pre-treatment respiratory** |  |  |  |  |
| 24-hours | 1.00 | - | - | - |
| Spontaneous | 7.000 (4.000-12.250) | **<0.001** | - | - |
| **Pre-treatment feeding** |  |  |  |  |
| Oral | 1.00 | - | 1.00 |  |
| Tube | 0.132 (0.067-0.261) | **<0.001** | 0.244 (0.117-0.505) | **<0.001** |
| Gastrostomy | 0.070 (0.034-0.144) | **<0.001** | 0.176 (0.080-0.391) | **<0.001** |
| **CHOP-INTEND score, at baseline** | 1.148 (1.104-1.193) | **<0.001** | 1.106 (1.061-1.153) | **<0.001** |

**OR:** Odds ratio, **CI:** confidence intervals. Bold values indicate statistically significant
(*p-value*<0.05). Omnibus test chi-square test=100.971, *p*-value<0.001; Hosmer and Lemeshow test=5.006,
*p*-value=0.757 and Nagelkerke R^2^=0.405.
