# Supplementary material for: Nusinersen for children with type I spinal muscular atrophy: 4 years’ clinical experience in Turkish cohort
Source: Front Neurol. 2025 Mar 27;16:1541507. doi: 10.3389/fneur.2025.1541507 (PMC11983886; doi:10.3389/fneur.2025.1541507)
Supplement: Supplementary file 7 [file Table_5.DOCX]

|  | **n (%)** |
| --- | --- |
| Mild Advers Event  Moderate Advers Event  Serious Advers Event | 33 (30.3%)  30 (27.5%)  46 (42.2%) |
| Gastrointestinal bleeding  Pneumonia  Acute Respiratory Failure  Local Edema  Respiratory Stress  Proteinuria  Sepsis  Thrombocytopenia  Atelectasis  Pyrexia  Short-Term Allergic Reaction  Aspiration Pneumonia  Hydrocephalus  Vomiting  Cough | 2 (1.8)  24 (22)  10 (9.2)  2 (1.8)  5 (4.6)  19 (17.4)  6 (5.5)  3 (2.8)  5 (4.6)  7 (6.4)  8 (7.3)  3 (2.8)  1 (0.9)  6 (5.5)  8 (7.3) |
| **Total** | **109 (100)** |

**Supplementary Table 5.** Adverse Events
