# Supplementary material for: Nusinersen for children with type I spinal muscular atrophy: 4 years’ clinical experience in Turkish cohort
Source: Front Neurol. 2025 Mar 27;16:1541507. doi: 10.3389/fneur.2025.1541507 (PMC11983886; doi:10.3389/fneur.2025.1541507)
Supplement: Supplementary file 8 [file Table_6.DOCX]

**Supplementary Table 6. Number of patients per cohort and time point.**

| **Cohort** | **Pre-Treatment** | **T6** | **T14** | **T26** | **T38** | **T46** |
| --- | --- | --- | --- | --- | --- | --- |
| **A** | 46 | 44 | 26 | 14 | 6 | 3 |
| **B** | 147 | 144 | 74 | 37 | 17 | 3 |
| **C** | 49 | 46 | 26 | 16 | 6 | 3 |
| **D** | 35 | 25 | 16 | 7 | 5 | 2 |
| **E** | 33 | 25 | 19 | 14 | 12 | 4 |
