# Supplementary material for: Nusinersen for children with type I spinal muscular atrophy: 4 years’ clinical experience in Turkish cohort
Source: Front Neurol. 2025 Mar 27;16:1541507. doi: 10.3389/fneur.2025.1541507 (PMC11983886; doi:10.3389/fneur.2025.1541507)
Supplement: Supplementary file 9 [file Table_7.DOCX]

| **Cause** | **n (%)** |
| --- | --- |
| Acute Respiratory Failure  Sepsis  Cardiopulmonary Arrest  Pneumonia  Aspiration | 30 (45.5)  12 (18.2)  9 (13.6)  8 (12.1)  7 (10.6) |
| **Total** | **66 (100)** |

**Supplementary Table 7.** Causes of Mortality
